# Symbolism and ritual practices related to hunting in Maya communities from central Quintana Roo, Mexico

**DOI:** 10.1186/s13002-015-0055-x

**Published:** 2015-09-29

**Authors:** Dídac Santos-Fita, Eduardo J. Naranjo, Erin I.J. Estrada, Ramón Mariaca, Eduardo Bello

**Affiliations:** Centro Regional de Investigaciones Multidisciplinarias (CRIM), Universidad Nacional Autónoma de México (UNAM), Av. Universidad s/n, Circuito 2, Chimalpa, Campus Morelos de la UNAM, CP 62210 Cuernavaca, Morelos México; Grant holder from the Posdoctoral Grant Program at UNAM, Centro Regional de Investigaciones Multidisciplinarias, UNAM, Cuernavaca, México; El Colegio de la Frontera Sur (ECOSUR) - Unidad San Cristóbal, Ap. 63, CP 29290 San Cristóbal de Las Casas, Chiapas México

**Keywords:** Ritual hunting, Ritual deposit, Carbine (firearm), Spirit/evil-wind *Sip*, Jaws, Yucatan Peninsula

## Abstract

**Background:**

Some Mayan peasant-hunters across the Yucatan Peninsula in Mexico still carry out a hunting ritual –*Loojil Ts’oon, Loj Ts’oon* or *Carbine Ceremony*– in which they renew the divine permission for hunting in order to continue deserving the gift of prey after a period of hunt. Thus they are granted access to game by the gods and the Lords of the Animals, particularly the spirit/evil-wind call*.* This paper focuses on the *acts* within the *Loojil Ts’oon* –which is performed in the X-Pichil community and surrounding area– that make it unique among the hunting rituals performed in other parts of the Peninsula.

**Methods:**

The *Loojil Ts’oon* hunting ritual was observed and registered in audiovisual format in two different occasions in X-Pichil (Friday 04/29/2011 and Friday 07/29/2011). Afterwards, we delivered digital videodisks (DVD) to hunters and their families and to the *j-men* (the magic-medic-ritual specialist) who participated in these ceremonies. This delivery produced confidence among participants to talk more openly and in-depth about the *Loojil Ts’oon*, revealing symbolic, psychological, and material details previously unknown to outsiders. Qualitative information was obtained through the ethnographic method using techniques such as participant observation and guided tours. Semi-structured interviews were carried out to obtain complementary information.

**Results and discussion:**

On one hand, we describe the preparation and cleansing of the “*Sip* soup”, as well as its parading and distribution –delivery to the spirit/evil-wind *Sip*– on the streets of the community (highlingting the role of the rooster as a counter-gift). On the other hand, the cleansing of the jaws (of deer: *Odocoileus virginianus*, *Mazama* spp.; and peccaries: Tayassuidae) and their return to the Lords of Animals in the hills so that they may give these animals new life.

**Conclusions:**

By performing the *Loojil Ts’oon,* the act of killing an animal is legitimized. The kill transforms into an exchange to perpetuate life, in which gods and Lords of animals grant the hunter the solicited new game if he has completed his ritual duties and has not broken the prescribed hunting rules. The *Loojil Ts’oon* does not only represent the continuity and regeneration of animals, that is, fauna as a resource, but also of the whole hunting cycle. The hunter does so to maintain and recreate order and equilibrium in one’s relationship with nature as a whole, with the rest of one’s social group, and with oneself. Thus, hunting transcends the exclusively material dimension of a subsistence activity.

## Background

Wild fauna has always constituted a significant historical and cultural element for humans around the world. Its value makes it subject to use and management practices which vary according to specific historical and geographical context [1–3]. Most wildlife resources are obtained through hunting. Defining and classifying hunting types do not properly depend on hunted species nor on biological/ecological criteria, but rather on the social, cultural, economic, and political context. In general terms, it can be said that there is a progressive trend towards commercial hunting in African tropical forests, while subsistence hunting prevails in Neotropical forests, with the exception of the Belen market in Iquitos, Peru [4]. On the other side, sport hunting remains a predominant practice in North America since its origins in the nineteenth century, while commercial hunting is now widely rejected and subsistence hunting is extremely rare [5].

In rural areas of the Neotropics, human groups continue to be greatly dependent on wild fauna as a source of nourishment, medicine, clothes, tools, ornament or ritual elements, and even income [6–11]. Specifically, recent studies have documented uses of over 60 species of wild animals by indigenous Maya^1^ inhabitants in the Yucatan Peninsula, southeast Mexico. In this paper, we use the term wildlife in reference to free-ranging large- and medium-sized terrestrial vertebrates, whose populations eventually may be kept in captivity. Among these animals are: white-tailed deer (*Odocoileus virginianus*), red brocket deer (*Mazama* spp.), collared peccary (*Pecari tajacu*)*,* white-lipped peccary (*Tayassu pecari*), paca (*Cuniculus paca*), nine-banded armadillo (*Dasypus novemcinctus*), coati (*Nasua narica*), pocket gophers (*Orthogeomys hispidus*), birds such as ocellated turkey (*Meleagris ocellata*; endemic of the Yucatan Peninsula), great curassow (*Crax rubra*) and black guan (*Penelope purpurascens*), and reptiles such as the red-eared turtle (*Trachemys scripta*), among others [13–17].

The main purpose of subsistence hunting is to satisfy the hunter and his family’s –and, occasionally, the community’s– basic needs. Subsistence hunters usually go hunting for food, although they might sell the surplus meat within their communities or other nearby communities. In contrast, commercial hunting is mainly motivated by the sale of prey for money [1, 17, 18]. However, basic needs are not only material, but also symbolic and religious. Therefore, hunting is not just a simple material practice, but rather a complex, way of obtaining resources from nature lying on a wide social, symbolic, and ritual construction of reality [19] (see examples for Amazonian groups in Reichel-Dolmatoff [20], Chaumeil [21], Rival [22], and Belaunde [23]).^2^

Rituals are rooted in the profound belief that human beings cannot live without making associations between their destiny and the natural and supernatural elements of nature. For the Maya, for example, gods, spirits, and other forms of hidden and mysterious forces still remain. These condition the lives and destinies of humans, either helping or opposing them –as adverse, dangerous, or simply bothersome beings– and ultimately determine the conducts of individuals and social groups [31, 32]. Essentially, in Mayan religion a sacred “contract of reciprocity” exists among humans and supernatural powers. This benefits humans in their daily work by providing protection, health, food, and other basic products in exchange for payment, often in advance [33]. These power entities must be recognized and remunerated for their favor and permission to make use of the forest –by means of ceremonies and religious acts– in order to maintain their favor and safe passage, or else avoid disaster [31, 34]. In other words, the respectful attitude captured in rituals is the result of prevailing fear of a danger filled, yet sacred nature. The remaining ritual practices in Mayan communities, such as those related to agricultural cycles –the *Ch’áa Cháak* (rain petition ceremony) and the *Janli Kool* (harvest thanksgiving)– exemplify the reciprocity-gratitude-dependence that exist in relation to the giver powers.

This is repeated in hunting, where the links between Maya people and wildlife use (especially deer and peccaries) demonstrate their beliefs, ritual practices, and cosmovision [31, 35–38], as it did in pre-Columbian times [39–43]. For the hunter to be able to find prey, he will have to temporally enter a supernatural domain and deal with certain divinities, forces, or spirits who, among other things, are the owners and guardians of animals and, by extension, of the forest itself [41]. There are several power entities corresponding to the “Lords of the Animals”. They are deemed the most ancient characters among all of the supernatural beings and spirits. Furthermore, they are omnipresent in the contemporary Mayan area and in other indigenous groups from Mesoamerica, among whom they have diverse names and representations [19, 43–47].^3^ Among their roles is the granting or denying of prey to the hunter, whose morality and conduct will be constantly tested. Among current Mayan communities in the Eastern half of the Yucatan Peninsula it is common to hear mention of the spirit/evil-wind called *Sip* as the main entity among the Lords of Animals. The fact that the *Sip* appears before the hunter is always a serious warning sign caused by the lack of compliance with the established “contract” and the prescribed rules for hunting. This entity is particularly associated with deer; when it manifests itself in front of a hunter it typically appears in the shape of this animal.^4^ Other mentioned power entities related to animals in this area are the *Yuum K’áax* (“Lord of the forest”), the *aluxes,*^5^ and more recently Saint Eustaquio and other catholic saints [36, 39, 51].

There are few studies that go in depth when describing and analyzing specific hunting rituals carried out by indigenous groups from Southern and Southeastern Mexico. The noteworthy exceptions are the works by Marianne Gabriel [36] with Maya from Yucatan, and those by Danièle Dehouve [19, 52] with Tlapanecs from Guerrero, Mexico. Recently, Santos-Fita [38] carried out a first comprehensive ethnographic approach to the *Loojil Ts’oon* –*Carbine* (firearm) *Ceremony*, or *Ceremonia de la carabina* in Spanish–, among Mayans from X-Pichil community (central Quintana Roo state). As part of said work, this paper focuses in highlighting those *acts* within the Ritual that make it unique, different even from other Mayan hunting rituals. The a) preparation and cleansing of the “*Sip* soup“ and its b) parading and distribution –delivery to the spirit/evil-wind *Sip*– on the streets (with a noteworthy role of the rooster as a counter-gift) are described. Furthermore, the c) cleansing of the jaws (of deer and peccary) and d) their return to the Lords of Animals, depositing them in the forest, are explored.

## Methods

### Study area

**Fig. 1:**
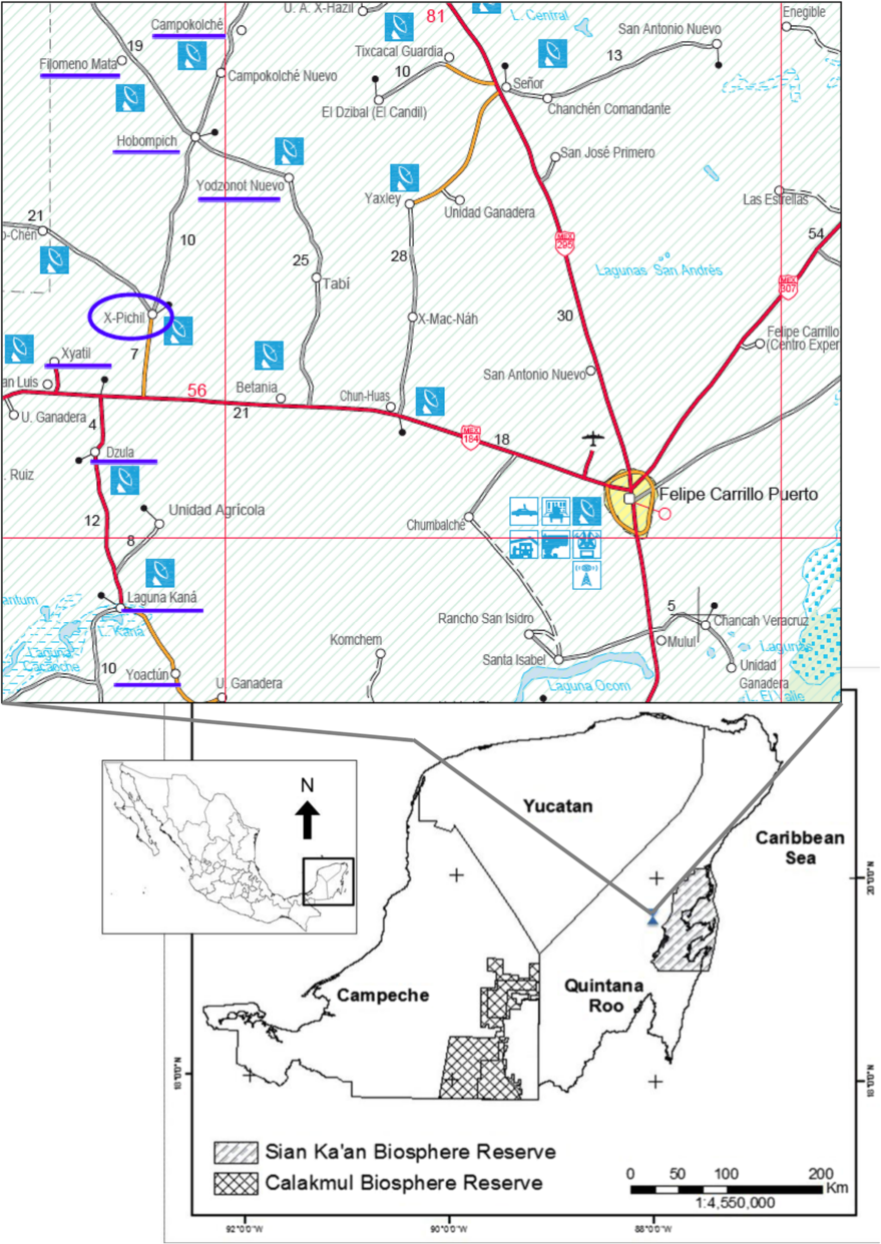
Location of X-Pichil and neighboring communities where the hunting ritual *Loojil Ts’oon* - *Carbine Ceremony* is performed in central Quintana Roo state, Mexico. Cartographic design by David Uribe Villavicencio. Map taken from Secretaría de Comunicaciones y Transportes, Mexico (2006)

The *Loojil Ts’oon* hunting ritual seems to be limited to a handful of Mayan communities in central Quintana Roo (a state located in the Yucatan Peninsula, Mexico), among which X-Pichil (pop. of about 2300; 40 km from the municipality’s administration center in Felipe Carrillo Puerto) stands out. Its inhabitants affirm that no hunter skips this Ceremony. The Ceremony is also celebrated in X-Yatil, Hobompich, Kampokolché, Filomeno Mata, Dzulá, Yoactún, Laguna Kaná, and Yodznot Nuevo (Fig. 1). However, according to people’s comments, hunters in these communities do not always abide by the obligation of performing the Ceremony.

The inhabitants of the study site are Maya who historically have called themselves *macehuales*, or descendants from rebels of the nineteenth century Cast War [53], who found refuge in the rainforests of the central-eastern Yucatan Peninsula. Their mother tongue is Yucatec Maya, but a few exceptions (elders), virtually all residents are fluent in Spanish. The immense majority of people in X-Pichil are catholic pertaining to the Maya Church established during the Cast War. This Church maintains a hierarchical structure inherited from the military movement [54]. Such religion is specific of the macehuales, for whom the Maya Church is an element of cultural identity [55]. The Maya Church constitutes an institution reflecting and regulating social life as a historic product of two religious complexes: Catholicism and Mayan traditional beliefs on agricultural practices linked to the Gods of Rain and other powers of Nature that prevailed after the Spanish Conquest (modified from Estrada [55], pp. 167–168).

The most important economic activities in X-Pichil are subsistence agriculture based on corn (*Zea mays*), beans (*Phaseolus* spp.), and squash (*Cucurbita* spp.), as well as commercial production of habanero pepper (*Capsicum* spp.), vegetables, honey, and small household businesses (grocery, stationery, car parts). Other relevant sources of income are transportation services, hammock and cloth making, and employment in the tourist industry in the city of Carrillo Puerto and the Maya Riviera. Hunting and fishing are often practiced as complementary food sources and occasionally for trade.

### Data collection and analyses

This work about the *Loojil Ts’oon* hunting ritual was part of a wider project aimed to know the *uses and custom* (*usos y costumbres* in Spanish) ruling wildlife use and management through hunting in Maya communities of the Yucatan Peninsula. Knowledge about *uses and custom* allows to distinguish norms followed by Maya people to hunt for subsistence, which in turn is useful to contrast local (Customary law) and national law systems including their environmental policies and institutional instruments for wildlife management (see Santos-Fita [38]).

This research was conducted using qualitative and quantitative techniques within an interdisciplinary approach. Qualitative information about the *Loojil Ts’oon* hunting ritual was obtained through the ethnographic method, using techniques such as participant observation and guided tours. Semi-structured interviews [56, 57] were applied to several hunters and the two *j-men* (generic contemporary Mayan name for a magic-medic ritual specialist) of X-Pichil to complement the information.

The Ceremony was observed and registered in audiovisual format in two different occasions in X-Pichil. The first of these occasions was in the home of Rufino Chuc Pool, 38 (Friday 04/29/2011). He and his son use a single carbine, which had helped them gather 15 jaws. Exactly three months later (Friday 07/29/2011) a second *Loojil Ts’oon* was registered by express request from Don Manuel Balam Coh, 53, a man regarded by the inhabitants of X-Pichil and its vicinity as the best and most prestigious *j-men*. This ritual was his two sons’ and brother-in-law’s. This joint ceremony was carried out in Don Manuel’s house and officiated by him; it included three carbines and 41 jaws.

As a first step to share our results with the community, we delivered digital videodisks (DVD) to hunters and their families and to the *j-men* participants in the two *Loojil Ts’oon* ceremonies that could be seen and recorded. This delivery produced confidence among people to talk more openly and in-depth about the hunting Ceremony, revealing symbolic, psychological, and material details previously unknown to outsiders. Finally, the software QSR N6® (QSR International, Pty Ltd 2002) allowed to capture, organize and systematize qualitative data for subsequent analyses.

## Results and discussion

### Why and how often is the ritual carried out?

«*Loojil Ts’oon is an offering for the previously hunted animals*» (Don Manuel Balam, *j-men* of X-Pichil; 2011)

Hunting is an essential activity for the *Loojil Ts’oon.* This ritual, correspondingly, is a regulatory and continuity mechanism for hunting. Its main objective is to renew the divine permission of hunting and thus continue to deserve the gift of prey. A hunter will obtain this if he keeps up his ritual commitments and obligations, that is, if he correctly performs the *Loojil Ts’oon* and does not break the rules of hunt as prescribed by the power entities in charge of the animals and the forest, particularly the *Sip.* Among these obligations the enablement for the revival of the hunted animals is prominent (see also Dehouve [19, 52], Brown [45], Brown and Emery [46]).

The carbine “keeps count” of the prey by the number of gathered (complete) jaws. It marks the cyclic pace of hunting periods and, in consequence, it defines the frequency of the Ceremony in order to start a new hunting cycle. Prey is assigned to it, not the hunter. To complete a cycle, the right number of hunted animals must be 13, taking only deer and peccary into account, even though a few additional individuals are a requisite for the Ceremony. During the second recorded Ritual the count of jaws corresponded to 13 jaws per hunter plus those corresponding to the game meat required for the Ceremony (41 jaws for three hunters in this case). In a way, the carbine is a mediator vehicle between the hunter, the Lords of Animals, and the prey they provide.

When the hunter/carbine completes his/its allowed time of hunt but he continues to go to the forest, he starts to receive warning signals from the Lords of Animals, such as: i) repeatedly running into poisonous snakes on roads or in the forest (always the first warning signs), ii) tripping and getting hurt with a bough or some other object, iii) having an *alux* throwing rocks at him to scare him and make the hunt more difficult, or iv) having an evil-wind (*mal aire* in Spanish) cause fever, headaches, or other physical or psychic discomforts. The warning signs may also come to the hunter in dreams. These calls to attention warn him that he is over his quota and that he must perform his *Loojil Ts’oon.* If he does not perform his obligations as a “good hunter” this person or his family members can receive a punishment, even one that is fatal. Thus, if a hunter perceives it is time for him to perform this Ceremony, even before any warning signs appear, he must do it without delay.

Contrastingly, if the hunter pays no attention to warnings and continues to go out to hunt without performing the Ritual, thereby violating the established “contract” and prescribed rules, the initial warning is replaced by a consequence, namely grave infirmity or even death. Thus, among Mayans, health is understood as a state of order and equilibrium between individuals or collectivities and the natural and supernatural world that surrounds them [58]. Becoming sick is a direct consequence of breaking or altering this harmony and is perceived as: «punishment for violation to the transactional code which rules the relations of an individual with both his community and [the power entities of] nature» (Bartolomé [35], p. 243).

“Winds” are one of the multiple manifestations of the gods and power entities. Among these, the “evil-winds”, which are actively punishing only on Tuesdays and Fridays, are related to the Lords of Animals who regulate hunting. As a consequence it is only on these days that they may be summoned to receive offerings. Thus, the *Loojil Ts’oon* may only be performed on either Tuesdays or Fridays –and the next day the hunter is required to go to the forest to give back the jaws–. Furthermore, the *j-men* can only perform Ritual-related prayers and cleansings (*limpias*^6^ in Spanish) in these specific days of the week. For the *Loojil Ts’oon* the presence of prejudicial, or potentially prejudicial supernatural powers, such as the *Sip,* is required. The risk and responsibility of the *j-men* is greater, since all the participants of the ritual and nearby people are exposed to damage from the evil-winds.

Contrastingly, on the rest of the week –Monday, Wednesday, Thursday, Saturday, and Sunday– other winds, deemed “good”, circulate. These days are proper for rituals directly related to the agricultural cycle, such as the *Janli Kool* or harvest thanksgiving. The *Ch’áa Cháak,* rain petition, is usually practiced on Saturdays.

In other Mayan communities there are hunting rituals that differ from the *Loojil Ts’oon* of the X-Pichil area. In the community of Xocén, Yucatan, for example, carry out the *Loj Ts’oon* ceremony: an offering of domestic meat and prayer dedicated to the *Sip* and other supernatural powers. They also perform a cleansing ceremony –*k’eex* (to change, to substitute)– for the hunters, their families, and their firearm, which is lain on the ground ([37]; Santos-Fita, unpublished data). Furthermore, LLanes-Pasos [59] speaks of a Mayan ritual called *Loj ts’on*, which is also carried out in Quintana Roo to re-consecrate the carbine after a certain number of hunted animals.

It can be appreciated that the Yucatec Maya terms to name all these rituals are basically the same. In spite of the considerable differences in structure, material objects, symbolism, and social structure around each individual ceremony in each community (see also Gabriel [36]), the purpose of the ceremonies is always the same: to renew the divine permission to hunt. The literal translation from the contemporary Yucatec Maya for these terms is either “hunting offering” or “hunter offering” [60]. Nevertheless, the townspeople from communities where it is common practice call it the “Carbine Ceremony”, as did their immediate forebears. This bears witness to the enormous value, both material and especially symbolic, that Mayans allocate to the carbine as a central part of hunting rituals. The presence and use of firearms, such as carbines (starting on the XVI Century with the arrival of Spaniards), eventually led to a reinterpretation of the ancient practices and rituals related to hunting.

### The ritual deposit as a nucleus of ceremonial practices in Mesoamerica

**Fig. 2:**
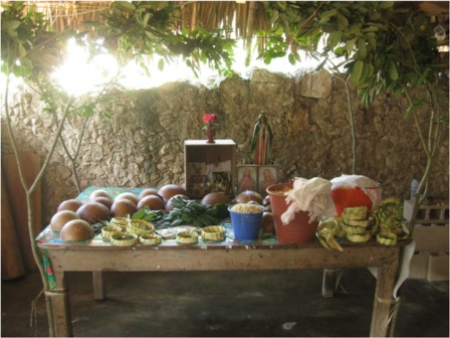
Ceremonial deposit, where most of the *acts* of the *Loojil Ts’oon* ritual occur. Source: photo by Dídac Santos-Fita; X-Pichil community, Quintana Roo (2011)

The *Loojil Ts’oon* ritual is structurally complex in its richness of material objects, prayers, and symbolisms. It contains and links elements of both Mayan cosmovision and catholic tradition and it involves diverse members of the community that might participate actively or not. Most of its *acts* occur around the sacred space. This space constitutes the so-called “ceremonial or ritual deposit” (*depósito ritual o ceremonial* in Spanish), which in the recorded Ritual consists of a table and the space that surrounds it (Fig. 2). According Danièle Dehouve (Pers. Comm., 2011), this concept implies that ritual practices of Mesoamerican indigenous peoples, from pre-Hispanic times and living on to this day, are composed of much more than simple or random acts. The expressions and manipulation of objects on the floor or on the furniture is not done by chance nor are these artifacts deemed simple gifts. These objects go well beyond the role of acting as offerings (from the Latin *offerenda*: things to be given). Additionally, this implies that sacrifice is not necessarily the central aspect of the Ritual, but rather another act within it [61]. Groups like the Chontal, the Mixe, the Nahua, the Totonac, and the Tlapanec, among others, also have their own ritual deposits as do the Mayans [61–64], which points to this as a defining and essential feature of the religious practices among indigenous groups within the Mesoamerican cultural area.

**Fig. 3:**
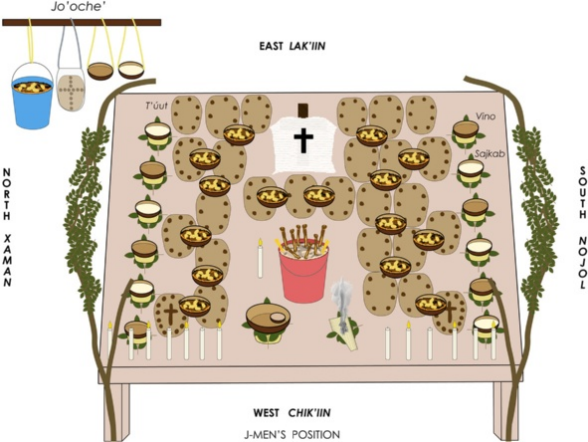
Layout of the objects in the ritual deposit during the food and drink offering: *t’úut* (ritual tortilla with pulverized squash seeds), mingled chunks of deer, peccary, and rooster meat, and *vino* and *sajkab* (ritual beverages). In addition, 13 candles (seven burning and six unburned) and incense (*pom*). Design: María Fernanda Nemer and Dídac Santos-Fita (DSF); Source: DSF’s fieldwork, 2011

All the material objects present in the ritual deposit of the *Loojil Ts’oon,* including those which are not part of the offering, are intentionally placed in defined numbers and quantity –“7”, “9”, and “13”, of capital importance within pre-Hispanic cosmovision; and “10”, understood as a half of “20” [35, 65, 66]–, as well as numerical series and a specific disposition by groups in both the horizontal and vertical planes (Fig. 3). Furthermore, this ritual deposit is a figurative recreation of the Mayan world and universe in its quadrilateral shape and having human beings within (for a more detailed description see Santos-Fita [38]; and also Villa Rojas [31], Sosa [67], Gabriel [68–70]).

### *Sip*-exclusive offering and the importance of the rooster as a counter-gift

The *Sip* requires a special offering, different from that of all the other gods and Lords of Animals. Furthermore, it does not receive it on the table (the *Sip* does not “sit” with the other power entities). Instead, it is the men who have to bring their offering to this Lord of Animals after performing a cleansing on it. In the words of Don Manuel Balam:

«*The evil-wind has a name, it is the Sip. It is a small deer, but with big antlers and they have wasps. It is the evil one, it does exist. […] It is also another name for the Spirit of Animals. If you have killed many, it becomes evil against you and attacks you, that causes the evil-wind. […] The one who actually punishes you if you go too far in your hunting. That is why we make the soup, it is a way to ask forgiveness from the spirits. It is food for the evil-wind. […] It is always set apart,* [not on the table], *that is why the first food is always for it, because it is the bad god, it punishes a lot*.» (2011)

**Fig. 4:**
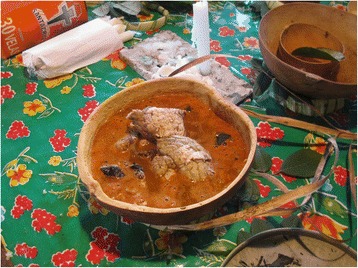
“*Sip* soup” (*jo’och Sip*): a special offering for the spirit/evil-wind called *Sip,* the main entity among the Lords of Animals. Source: photo by Dídac Santo-Fita; X-Pichil community, Quintana Roo (2011)

In a large squash-pot (in the recorded event *Lagenaria siceraria*, Cucurbitaceae) with a ring and stripes made of reed so it can be suspended in the air, Don Manuel puts (in this order) the ingredients of the “*Sip* soup” (*jo’och Sip*): i) an orange broth which is made of the foam previously obtained in the cauldron by parboiling and seasoning the game (deer and peccary) and rooster meats; ii) a special tortilla in pieces, called *péenkuch,* which is cooked buried in the ashes of the fireplace (*k’óoben*); iii) the cut up brains and parts of livers (*t’uup taman*) of the game and the rooster to be offered; and iv) 13 dried and ground *sukure* peppers (*Capsicum* spp.; also called *socorro* in other communities of the region) (Fig. 4).

During the *Loojil Ts’oon* ritual, the order of the cleansings the *j-men* performs is: i) the “*Sip* soup”, ii) the jaws, carbine(s), and backpack(s), and, finally iii) the hunter(s). In front of the table, always looking toward the East, Don Manuel Balam raises the soup squash-pot while moistening a group of 9 twigs of *sip che’*^7^ in alcohol mixed with *tancasche’*^8^ bark shavings and 9 dried and ground *sukure* peppers. This cleansing lasts a little over seven minutes of prayer.

The fact that this soup is the single offering within the Ritual that is cleansed is noteworthy. This raises some questions such as: as the soup is cleansed is it being simultaneously delivered so that the evil-wind will be expelled? Or has the soup accumulated evil-wind during its preparation and thus must be cleansed before its presentation? It must be pointed out that in Mesoamerica, ritual cleansings are always simultaneously acts of expulsion, purification, and protection (D. Dehouve, Pers. Comm., 2011). A third option is looking for the answer to the need to cleanse the “*Sip* soup” in its ingredients, as a symbolic vehicle for giving back in its gift and counter-gift sense. This would explain the presence and fundamental role of the rooster in this Ceremony.

During the *Loojil Ts’oon* it is not until night has properly fallen (once it is dark) that the soup is actually taken to the *Sip*. Prior to this, the rest of the food and drink are offered to all the other power entities that are invited to sit around the table (see Fig. 3). These are 13 in total and they include catholic saints, nature personifications, and divinities of pre-Hispanic Maya origin, two of which have a virgin status (catholic concept), but they do not include the *Sip* (for further detail see Santos-Fita [38]).

The *j-men* delivers the squash-pot with the cleansed soup to two helpers. They exit the house and take the right hand street. In almost complete darkness, they complete a walk on foot through the streets always taking left turns (counterclockwise), until they reappear on the opposite side of the street on which they started. During their walk the *j-men*’s helpers spill some “*Sip* soup” on the ground and throw a twig from the *sip che’* bouquet, which was previously used for the cleansings, every few steps. They cross themselves as they advance. Finally, they stop about fifteen meters from the entrance door to the house in which the Ceremony is being performed. At this point, the *j-men* receives them to cleanse them of any evil-winds they might have accumulated during their parading before they can safely approach the ritual deposit.

This ritual *act* is explained as a simultaneous expulsion and return. One of the key aspects of the *Loojil Ts’oon* ritual is the use of rooster (its brain and liver) as a counter-gift domestic animal. The prey that is delivered to the hunter is a gift from gods, from the *Sip,* and from other Lords of Animals. The way in which the hunter is required to pay back these gifts of wild meat is the offering of rooster, a domestic counter-gift. The interesting part of this reciprocity of gifts and counter-gifts between the Lords of Animals and humans, is perfectly depicted in the “*Sip* soup”, which includes in its ingredients both wild and domestic animal products. In the words of Don Manuel Balam:

«*The rooster is the offering for the Lords of Animals. It is an exchange. You trade the rooster for the wild animals. […] God takes it, it takes the rooster’s spirit. […] It is said that when the prayer comes to an end they take it and if they want to punish you, you can hear a rooster screech when you’re out in the forest or at night. If the rooster sings, the Sip is telling you that punishment is coming, it is giving you warning. […] If something goes wrong in the Ceremony it is not received, then* [the hunter] *will hear many noises in the forest or in his milpa* [*maize field*]*, it is the sign that there will be a punishment. […] It helps the Sip give warning. If you do not realize and do not heed this warning, the Sip may come before you. […] The singing rooster is like a spirit, it is the same one offered in the soup. It does not sing here in town, it sings out in the forest*.» (2011)

The justification for the use of rooster (its spirit) in particular as a counter-gift lies in its symbolic, and not material, value. The *Sip* will use the power of rooster’s song to manifest itself, communicating to the hunter that the Ceremony has not worked due to a liturgical error of some sort. This must be swiftly corrected by repeating the whole Ritual. The significance of the rooster is that, as a domestic animal from the domain of humans, the hunter must deliver it has an offering for the *Sip* to later use it as an instrument of warning (thus it can never be a hen). Considering the rooster was introduced by Spaniards to Amerindian lands by the late XV century, surely its morning call was noteworthy to the Mayans and, within the *Loojil Ts’oon,* it was translated as a call of warning.

### Returning the jaws for the rebirth of the hunted animals

It is not exclusive to the *Loojil Ts’oon* ritual that the hunter must return a part of the prey’s bones or their entirety (material restitution) to its owners and guardians once the Ceremony is concluded (symbolic restitution) so that they will bring new life to them. This ensures ritual efficacy to the renewal of the hunters’ divine license to hunt. Thus, a cycle is completed and a new one may begin only if the hunter promotes and facilitates the rebirth of the dead animals. Several studies in the Mesoamerican region register bone deposits in caves where it is typical to observe a mixture of remains of diverse hunted species [19, 45–47, 52, 72, 73]. There are also examples from the region north of Mesoamerica, such as the works of Neurath [74] with Huicholes and Alvarado [75] with Mexicaneros.

**Fig. 5:**
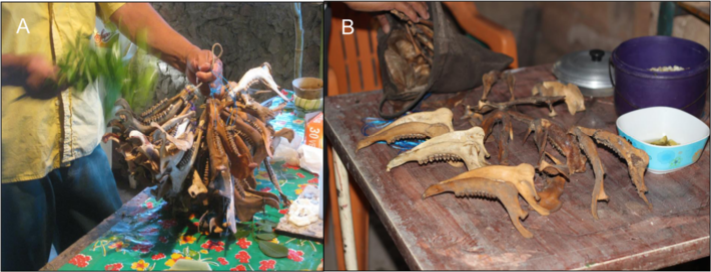
As an important part of the *Loojil Ts’oon*: (**a**) the *j-men*, in his role as a ritual specialist, cleanses the prey’s jaws (only deer: *Odocoileus virginianus*, *Mazama* spp.; and peccaries: Tayassuidae), (**b**) which the hunter has kept during the allowed hunting period in order to ritually remove the evil-wind (*mal aire* in Spanish) they have accumulated in death and thus return them in a proper state to their owners and guardians in the forest. Source: Photos by Dídac Santos-Fita; X-Pichil community, Quintana Roo (2011)

**Fig. 6:**
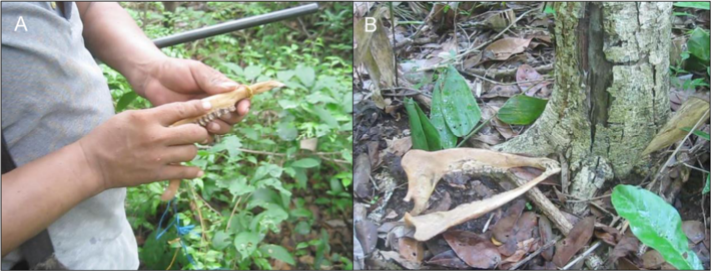
The day after the *Loojil Ts’oon* is performed, the hunter must: (**a**) go to the forest to deposit, that is, give back the jaws [larger in (**b**)] so that the Lords of Animals give them new life, thus completing the cycle and renewing the hunting permission as established by the supernatural entities. Source: Photos by Dídac Santos-Fita; X-Pichil community, Quintana Roo (2011)

Thus, it is noteworthy that the Mayan hunters in X-Pichil and other surrounding communities, who perform the *Loojil Ts’oon,* save and cleanse only the jaws of their prey (Fig. 5a and b) (for jaws saving, see also Valeri [29] or Ellen [76] for hunters from Southeastern Asia). Furthermore, when these bones are brought back to the Lords of Animals, they are deposited in the forest, not in caves (Fig. 6a and b) (see also Reyes [77], for an example with Nahua hunters from Ichcatepec, Veracruz, Mexico; Hamayon [25, 26] for Siberian hunters or Tanner [27] for Cree hunters from Canada). In the words of the two *j-men* of X-Pichil and two hunters:

«[The jaws] *must be taken because the offering, that is, the Ceremony is done. […] Because they* [the Lords of the Animals] *already know they are due this offering. […] The hunter must go to the forest to place the jaws there to give them new life so that they do not run out. This is the reason for the Ceremony, so that we will not run out of wild animals. So that the animals will continue to reproduce.*» (Don Nazario Chuc, *j-men* of X-Pichil; 2011)

«*When the ancient animals come and grab* [the jaws] *and take them. […] A new one grows, their body is put back on. I have prayed for it. I have done everything, now its body must come back. When it was killed it has a body, but now, since I have performed prayer on it, it has to take its own body.*» (Don Manuel Balam, *j-men* of X-Pichil; 2011)

«*Out there all the killed animals live again. […] There are some people who say everything runs out, but because of God it doesn’t. With God’s blessing, it does not run out. The killed animals don’t run out, they come to life again.*» (Don Manuel Balam, *j-men* of X-Pichil; 2011)

«*That is the tradition that our grandfathers left us. In ancient times it was believed that these bones* [jaws] *left in the forest will regain life. The j-men says he has confirmed it, after three or four days the bone that was deposited is no longer found. […] I am sure these bones regain life.*» (Marcelino Mis, hunter from X-Pichil; 2011)

«*My grandfather told me that when you give the jaws back to the wildernes it is like reviving what you have killed. […] These are ancient beliefs of the people who lived here. […] The jaws are not found after.*» (Rufino Chuc, hunter from X-Pichil; 2011)

While the Ceremony in itself is over, failing to give back the complete cleansed jaws the next day –Wednesday or Saturday– would render the Ritual incomplete by making the renewal of animals impossible. This would, additionally, leave the hunter at the mercy of the protect or entities for punishment. Thus, to complete the purpose of the Ritual, the hunter goes to the forest, depositing the jaws as he walks by while he looks for new prey. His permission and new hunting cycle are already valid since the previous day as long as he does not discover (by hearing the rooster’s song, for example) that liturgical errors, which would void the whole process, have been made. The correct way of placing the jaws is doing so on the west-looking side (*chik’iin*) of the trees, upon the ground and respecting some distance between each piece. The reason for this is that: «*When they come looking for it, they will walk in that direction.*» (2011). Don Manuel Balam also points out that it is preferable to place them at the foot of trees which are located in a *kalap*, that is, between two hills, because animals usually pass places like this and, in consequence, so do their Masters.

The fact that only the jaws of deer and peccaries are accounted for and given back to their Masters stands out. We are none the wiser as to why it is exclusively these two vertebrate groups, and none of the other commonly hunted species in the area which provide a considerable amount of nourishment such as the paca or large birds like the ocellated turkey or the great curassow (since birds do not have jaws, other bones could be used), are considered in this count. It does not seem the choice of ceremonial species is due to the knowledge hunters have of their biology, ecology or conduct, nor to the specialized way (techniques, instruments, social organization, temporal and spatial aspects of the hunt) in which they are obtained. The hunters have just as refined knowledge of several other species that do not receive this ritual treatment [13–15, 17]. Consequently, it is through considering the symbolic aspects of these species, and not focusing on their material significance, that we may be able to shed light on this question.

## Final considerations

To the Mayans there is a shared structural and symbolic base to any kind of ritual. However, in the *Loojil Ts’oon* or *Carbine Ceremony*, as it is performed in X-Pichil and other close by communities, there are unique *acts* related to hunting which are distinctive even from other hunting rituals performed in different Mayan communities in the Yucatan Peninsula [36, 37, 59].

The *Loojil Ts’oon* ceremony, just as any other hunting ritual, counters the risk of a hunter entering a non-human medium which is unpredictable and threatening in which subsistence means killing beings that are somehow considered superior to humans [41]. Thus, by performing the *Loojil Ts’oon* the act of killing an animal is made legitimate by creating a social and moral structure of action, emotion and control that ensures the hunter is acting correctly before, during, and after the hunt (*sensu* González [78]). In the words of a hunter, after performing his Ceremony, and Don Manuel Balam:

«*I feel safer, better protected, because I’m no longer carrying around the guilt for all the killing I have done, that is, for the animals I killed. Well, right now I feel good as new, the evil-winds I carried from this animals are all taken. […] Now I feel clean so I can look for others.*» (Rufino Chuc, hunter from X-Pichil; 2011)

«*What we have done now is saving the lives of others* [hunters]*.*» (Don Manuel Balam, *j-men* of X-Pichil; 2011)

Hunting transcends the role of an exclusively utilitarian and materialist subsistence activity. The act of killing is transformed into an exchange to perpetuate life, in which the gods and Lord of animals (as intermediaries and judges) grant the hunter the new solicited game once he’s complied with his ritual duties and has not broken the prescribed hunting rules. In particular, compliance with the Ritual constitutes an ideological element that integrates hunt in a legitimizing context that allows its reproduction. This is due, among other things, to the fact that this process is completed with the rebirth of the prey (the conservation and perpetuation of used species): «It can be viewed as a cycle in which flesh is reduced to bone and bone is regenerated as flesh.» (Braakhuis [44], p. 395). While there might be signs that this exchange might imply a relationship between hunting and sexuality, as has been observed among Mayan hunters in Yucatan (M. Gabriel, Pers. comm., 2011), a deeper approach is needed to determine whether this is the case for Mayan Yucatecs (not exclusively from central Quintana Roo). Furthermore, several specialists suggest a generalized idea that exchanges between humans and Lords (spirits) of animals constitutes a metaphorical “marital bond”. This is supported by ethnographic studies of indigenous peoples depending partially or totally on hunting throughout the world and by ethnohistoric evidence [19, 20, 24–28, 43, 44].

Furthermore, the *Loojil Ts’oon* does not only represent the continuity and regeneration of animals, that is, fauna as a resource, but also of the whole hunting cycle. Whoever performs the Ceremony does so to maintain and recreate order and equilibrium in one’s relationship with nature as a whole –as one’s destiny is linked to the care of nature, in order to survive–, with the rest of one’s social group, and with oneself. A “good hunter” will be favored by the supernatural powers, who will act as givers for him. Meanwhile, as hunting requires this symbolic construct which transforms an act of predation and appropriation into an agreed reciprocity with gods and spirits that protect animals (stressing the role of the rooster in this case) by means of ritual practice, it highlights the underlying sociocultural processes legitimizing the use and control of the physical spaces where there is interaction with wild fauna. That is, in those spaces in which the hunt occurs, ritual practice and regeneration of prey are socially constructed and recognized, thus constituting a territorial element and recreating an identity and a sense of belonging to this territory. Therein lies the present and future significance of ceremonies like the *Loojil Ts’oon*.

## Footnotes

1 Throughout this work the term *Maya* refers in particular to the ethnolinguistic group of peninsular or Yucatec Maya, that is, the inhabitants in Yucatan Peninsula, southeast Mexico [12].

2 In other parts of the world, there are interesting examples from Siberia [24–26], North America [27, 28], Southeastern Asia [29], and Northwestern Europe [30].

3 On the other side, there are other Lords of the Animals across the Amazon Basin, such as the *Curupira* and the *Caipora* (Brazil), the *Chullachaqui* or *Shapishico* (Peru), the *Tanamudak* (Shiwilu people; Peru), and the *Amasanga* (Kichwa people, Ecuador) [23, 48]. In addition, in the Andean region are known the *Pachamama*, and the *Coquena*: shepherd and protector of vicuñas (*Vicugna vicugna*) [49].

4 According to Mayan folk tales, from Pre-Hispanic times to date the spirit/evil-wind manifests itself to hunters in the shape of a deer. Usually, this deer is a short adult with great, branched antlers in which a wasp nest (that may attack the hunter) is carried [31, 32, 39, 50]. This contributes to the enormous significance of deer in mythology and magic in Mayan cosmovision, as it is frequently associated with the Lord of animals [41, 50], and carries out this protective role.

5 A name given to a mythological being, from the tradition of some Mayan peoples from the Yucatan Peninsula and Guatemala. Traditinally, *aluxes* (or *aruxes* in Quintana Roo) are generally invisible or have a wind form, but they may also take a physical appearance (dressed in Mayan attire) in order to scare humans or help them. They are associated to natural spots such as forests, caves, rocks, water, and cultivated spaces. It is believed that these mischievous beings are the incarnation of the Pre-Hispanic clay figurines that are abundantly found in crops across the Peninsula [31, 32].

6 The term *limpia* depicts a therapeutic practice of body cleansing with branches and leaves of diverse plant species [35].

7 *Bunchosia swartziana*, Malighiaceae [71]. Among the Maya people, some plants with magic-religious values that are used in ceremonies have onomastic relationship with ancient deities: S*ip che’* means “the *Sip* tree” [71] (see also Villa Rojas [31]).

8 *Zanthoxylum fagara*, Rutaceae [71].
